# Genotype and phenotype data standardization, utilization and integration in the big data era for agricultural sciences

**DOI:** 10.1093/database/baad088

**Published:** 2023-12-11

**Authors:** Cecilia H Deng, Sushma Naithani, Sunita Kumari, Irene Cobo-Simón, Elsa H Quezada-Rodríguez, Maria Skrabisova, Nick Gladman, Melanie J Correll, Akeem Babatunde Sikiru, Olusola O Afuwape, Annarita Marrano, Ines Rebollo, Wentao Zhang, Sook Jung

**Affiliations:** Molecular and Digital Breeding, New Cultivar Innovation, The New Zealand Institute for Plant and Food Research Limited, 120 Mt Albert Road, Auckland 1025, New Zealand; Department of Botany and Plant Pathology, Oregon State University, Corvallis, OR 97331, USA; Cold Spring Harbor Laboratory, 1 Bungtown Rd, Cold Spring Harbor, New York, NY 11724, USA; Department of Ecology and Evolutionary Biology, University of Connecticut, Storrs, CT, USA; Institute of Forest Science (ICIFOR-INIA, CSIC), Madrid, Spain; Departamento de Producción Agrícola y Animal, Universidad Autónoma Metropolitana-Xochimilco, Ciudad de México, México; Centro de Ciencias de la Complejidad, Universidad Nacional Autónoma de México, Ciudad de México, México; Department of Biochemistry, Faculty of Science, Palacky University, Olomouc, Czech Republic; Cold Spring Harbor Laboratory, 1 Bungtown Rd, Cold Spring Harbor, New York, NY 11724, USA; U.S. Department of Agriculture-Agricultural Research Service, NEA Robert W. Holley Center for Agriculture and Health, Cornell University, Ithaca, NY 14853, USA; Agricultural and Biological Engineering Department, University of Florida, 1741 Museum Rd, Gainesville, FL 32611, USA; Federal University of Agriculture Zuru, PMB 28, Zuru, Kebbi 872101, Nigeria; University of Lagos, Nigeria; Phoenix Bioinformatics, 39899 Balentine Drive, Suite 200, Newark, CA 94560, USA; Universidad de la República, Uruguay; National Research Council Canada, 110 Gymnasium Pl, Saskatoon, Saskatchewan S7N 0W9, Canada; Department of Horticulture, Washington State University, 303c Plant Sciences Building, Pullman, WA 99164-6414, USA

## Abstract

Large-scale genotype and phenotype data have been increasingly generated to identify genetic markers, understand gene function and evolution and facilitate genomic selection. These datasets hold immense value for both current and future studies, as they are vital for crop breeding, yield improvement and overall agricultural sustainability. However, integrating these datasets from heterogeneous sources presents significant challenges and hinders their effective utilization. We established the Genotype-Phenotype Working Group in November 2021 as a part of the AgBioData Consortium (https://www.agbiodata.org) to review current data types and resources that support archiving, analysis and visualization of genotype and phenotype data to understand the needs and challenges of the plant genomic research community. For 2021–22, we identified different types of datasets and examined metadata annotations related to experimental design/methods/sample collection, etc. Furthermore, we thoroughly reviewed publicly funded repositories for raw and processed data as well as secondary databases and knowledgebases that enable the integration of heterogeneous data in the context of the genome browser, pathway networks and tissue-specific gene expression. Based on our survey, we recommend a need for (i) additional infrastructural support for archiving many new data types, (ii) development of community standards for data annotation and formatting, (iii) resources for biocuration and (iv) analysis and visualization tools to connect genotype data with phenotype data to enhance knowledge synthesis and to foster translational research. Although this paper only covers the data and resources relevant to the plant research community, we expect that similar issues and needs are shared by researchers working on animals.

**Database URL**: https://www.agbiodata.org.

## Introduction

Genotype-to-phenotype (G2P) integration is the process of linking genetic data to measurable qualitative and quantitative phenotypes and traits. Historically, linking genetic markers or genes with desirable traits has led to improved cultivars with higher yields and quality, enhanced disease resistance or climate resilience. In the past two decades, the generation of high-throughput omics or ‘big data’ including plant genomes and pangenomes; genetic variation data including single-nucleotide polymorphisms (SNPs) and structural variations (SVs) including transcriptomes, phenotype, proteomes and metabolomes has changed the scale and scope of data analysis, knowledge synthesis and its application in translational research (1, 2). In addition, researchers and breeders worldwide have collected classic mutant phenotype and trait data, and more recently, there has been a substantial surge in the generation of large-scale phenotype data. Often, specific big datasets are generated by projects and analyzed to address knowledge gaps, but they often remain underutilized for discovering new knowledge. Although it is challenging to survey or predict which scientific data will be reused, by whom and for what purpose (3–5), several studies have shown that data reuse is generally limited (6–8). Researchers tend to primarily employ their own data for hypothesis testing, only occasionally incorporating other’s data for baseline purposes (9, 10). Going forward, the different data types produced in various experiments can be reutilized to synthesize new knowledge and to develop data-driven hypotheses for experimental research (11). However, integrating large-scale datasets from diverse sources can be challenging (4, 8) and typically involves infrastructure to support data sharing, data quality check, data re-formatting, curation and re-analysis (12–14). For example, genotype, phenotype and expression data for the same plant accessions were generated from various projects over a decade, each using inconsistent sample identifiers and different plant growth environments. Before utilizing these various datasets to investigate the genetic and environmental factors influencing a particular phenotype, establishing consistent sample names, gene IDs and phenotypes across all datasets will be needed and possibly require modification in the original data format. The fulfillment of the unprecedented potential of big data depends on the data being Findable, Accessible, Interoperable, and Reusable (FAIR) (15–17). To meet the FAIR standards, any dataset should include metadata (18, 19) providing the standard terms and details necessary for data interpretation using controlled vocabularies. Data and metadata standardization can be achieved by developing common community standards of data formats and descriptions so that diverse datasets from different sources can be accessed and interoperated for visualization and knowledge synthesis. A clear, organized and consistent method of capturing and exchanging agricultural data will ensure easier data discovery, comparisons and reuse by various stakeholders.

**Figure 1.**
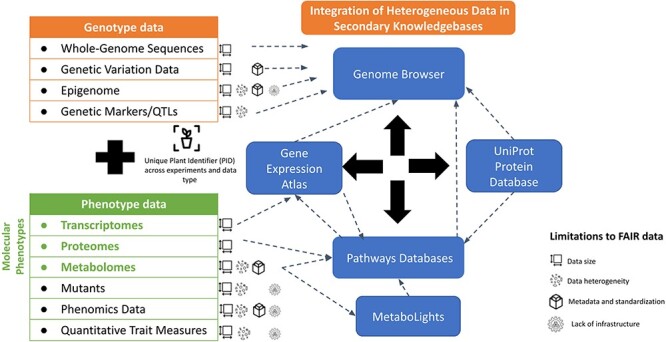
Current status of genotype-to-phenotype data integration. The left side illustrates the diversity of genotype and phenotype data. The right-hand side lists examples of the existing databases and knowledgebases which support the integration of heterogenous data types and their visualization.

Making data FAIR requires concerted efforts and communications among all parties involved in data generation and curation. In 2015, the AgBioData Consortium (https://www.agbiodata.org) was formed to identify and promote the means to consolidate and standardize common genomic, genetic and breeding (GGB) database tools and operations, with the goal of increased data interoperability for future research (16). At present, AgBioData comprises over 40 GGB databases and more than 200 scientists, fostering collaborations and open discussions about the common practices, challenges and solutions related to big data generated by agricultural researchers. AgBioData consortium has previously identified challenges facing GGB databases and suggested common guidelines for biocuration, ontologies, metadata, database platforms, programmatic access to data, communication among various partners and stakeholders and sustainability of genomic databases (16). AgBioData aims to (i) identify and address data-related issues by defining community-based standards, (ii) expand the network by involving all the stakeholders of the agricultural research community, (iii) develop educational material to train current and future scientists on database usage and the FAIR principles and (iv) develop a roadmap for a sustainable GGB database ecosystem. As part of this National Science Foundation Research Coordination Networks project, various working groups were established to address significant data-related challenges and requirements. One such group, known as the Genotype-Phenotype working group, was formed in November 2021 with the goal of identifying current challenges in annotating and integrating large-scale genotype and phenotype data. In this study, the Genotype-Phenotype working group summarizes common genotype and phenotype data types, repositories and knowledgebases, and the present state of FAIR practices of genotype and phenotype data (as illustrated in Figure 1). This paper provides (i) a brief introduction of the diverse data types and how they are generated, (ii) primary and secondary data repositories and databases for these data types, (iii) requirements of associated metadata and the minimum standards, (iv) examples of reuse and re-analysis of omics data and (v) limitations of data reuse. Finally, based on our surveys, reviews and community discussions, we list our recommendations that can provide the needed support to the plant genomic communities in making genotype and phenotype data interoperable and reusable for knowledge synthesis and fostering translational research needed for long-term agriculture sustainability.

## Genomics and transcriptomics data

### Whole-genome and transcriptome sequences

In the past decade, sequencing technology has evolved rapidly from the early days of time-consuming Sanger sequencing to high-throughput massive parallel sequencing that started the era of whole-genome sequencing (WGS) and transcriptome sequencing (20–22). There are three general methods of DNA/cDNA sequencing: (i) Sanger chain termination sequencing and Maxam Gilbert sequencing (23, 24); (ii) short-read sequencing known as next-generation sequencing (NGS) (25–28) including Ion Torrent, Solexa/Illumina and Roche/454 pyrosequencing (29) and (iii) more recent long-read Third Generation Sequencing (3GS) (30–34). Primarily, single-molecule real-time sequencing from Pacific Bioscience (PacBio) and nanopore sequencing from Oxford Nanopore Technologies (ONT) are examples of the 3GS. Illumina is currently the dominant and most popular platform in NGS for both genome and transcriptome sequencing because of its high accuracy, low cost and global distribution of its solutions for sequencing. PacBio and ONT are also gaining popularity and becoming more affordable for high-quality long-read/full-length sequences. Similarly, DNBSeq from MGI Tech, a subsidiary of the Beijing Genomics Institute group, and Ion Torrent Systems are making advances (35, 36). Several file formats are used in WGS, and the most common is the compressed FASTQ format (37) that is used for both NGS and 3GS sequencing. The original file formats for 3GS include legacy h5 format for PacBio (http://files.pacb.com/software/instrument/2.0.0/bas.h5%20Reference%20Guide.pdf), the industry-standard Binary Alignment Map (BAM) format (38) and the FAST5 format for ONT (39) that is based on the hierarchical data format (HDF5) (40) used for ONT data storage sequencer. Meanwhile, there are numerous basecallers available for conversion to FASTQ format (35) (https://long-read-tools.org/); in general, we find that sequencing data have achieved standard data formatting. Uniformity among available basecallers is essential because they play a critical role in converting data from various technologies into a standardized format, such as FASTQ. This uniformity facilitates data exchange, analysis and collaboration in the field of genomics and bioinformatics.

### Genome sequencing strategies for genotyping

Genotyping technology is a crucial component in linking genotype to phenotype. The process entails the generation of genetic profiles, which comprise DNA fragments, markers or sequences. These profiles serve to differentiate between different accessions, cultivars or siblings within a population in the initial stage. Subsequently, a correlation analysis is conducted to assess the relationship between the genotype profile and the phenotypic traits. The first-generation genotyping marker was Restriction Fragment Length Polymorphisms, which relied upon underlying differences in base pair sequences to create fingerprints after DNA regions were digested with known restriction enzymes (36). Later, the technological advancements led to genotyping by scoring microsatellite markers, simple sequence repeats or short tandem repeats (37). High-throughput low- and high-density SNP arrays (38) provide a cost-effective genotyping solution for studies such as population structures, genomic diversity, gene discovery and molecular breeding (39–42). Furthermore, development of whole-genome arrays made it possible to genotype a large number of samples in a short period of time, and data analysis simpler. However, designing an efficient array with high-quality SNPs for a particular crop usually requires significant investment upfront. As genome sequencing has advanced even further, researchers can now achieve whole-genome profiling through lower- or higher-coverage sequencing strategies such as NGS and 3GS. In essence, researchers now could choose from the various available options depending on their research goals and available budget. For example, sequencing of sub-sampled loci (43) has been widely used in phylogenomics studies for cost-effective large-scale genotyping. Skim sequencing (44) is another low-coverage WGS approach. Target enrichment sequencing investigates specific genomic elements via pre-defined probe sequences (45). Exome sequencing is a common type of target sequencing that focuses on protein-coding regions of genes (46). Amplicon sequencing is a highly targeted approach addressing specific genome loci. Genotyping-by-sequencing (47, 48) and restriction site–associated DNA marker sequencing (49, 50) are two popular, cost-effective sequencing strategies for shearing the genome via restriction enzyme(s).

The advent of high-throughput sequencing has generated immense amounts of data that are being used to capture intraspecies and interspecies genetic diversity and allow exploration of genetic variations. Regarding genotyping data structure, the 1000 Genomes project (https://www.internationalgenome.org/) spearheaded the first Variant Call Format (VCF) for standardizing the SNPs, indels and SV between two or more genomes at a given locus (51). The VCF has become the go-to format for variant data and associated metadata; over time, modifications of the base VCF file have expanded to include experiment-specific modifications, such as genome-wide association study (GWAS)-VCF (52) and genomic variant call format (tinyurl.com/5f8wpmhr), and accommodate variant information of polyploid genomes. In addition to the low-coverage genome sequence, transcriptome sequences are routinely used for genotyping and identification of useful genetic markers. More recently, the integration of single-cell genome sequencing and single-cell transcriptome sequencing tools has facilitated quantifying genetic and expression variability between individual cells (16). Like sequence data, genotyping data has standardized formats.

### Public repositories for genomic and transcriptomic data

Regardless of the sequencing platform or strategy used, raw sequencing data in compressed fastq.gz format are submitted to a public data repository such as National Center for Biotechnology Information (NCBI) GenBank, Sequence Read Archive (SRA) (53, 54) (Leinonen *et al.*, 2010; Kodama *et al.*, 2012) and/or Gene Expression Omnibus (55–57) via the NCBI submission portal. NCBI provides BioSample metadata templates based on organism lineage validation. Besides NCBI, the data can be submitted to the DNA DataBank of Japan (DDBJ) (58–60), SRA via the DDBJ submission navigation website or the European Nucleotide Archive (ENA) through the BioStudies portal. DDBJ, ENA and NCBI GenBank (Table 1 and Supplementary Table S1) form the International Nucleotide Sequence Database Collaboration (INSDC) (61, 62) and exchange data daily. Prior to publishing the results, all the life science journals require authors to submit their raw sequence data to the public INSDC repositories—a key component of the data sharing policies in the community of biologists (63). Additional public platforms that host the sequence data include the US Department of Energy Joint Genome Institute (JGI) (64, 65) that makes sequencing data generated by its collaborating projects available immediately to registered users and then follows public release on JGI and NCBI/SRA or GeneBank after a one-year embargo period. JGI also provides Phytozome (66), the Plant Comparative Genomics portal, for genome accessing, comparison and visualization (Table 2). *Nature* and *Scientific Data* request that sample metadata is deposited in one of the INSDC BioSample databases in conjunction with sequence data. It is crucial to use the standardized metadata at both the study and sample level to facilitate the curation and processing of transcriptomics data in a FAIR-compliant way. A few sequence repositories such as Zenodo (https://www.zenodo.org), DRYAD (https://datadryad.org), Figshare (https://figshare.com) and Harvard Dataverse (https://dataverse.harvard.edu) accept data submission in any file format.

**Table 1.** A list of active, maintained and updated public repositories for genomic, genotyping and transcriptome data

Database name	NCBI	DRA	ENA	GSA	IBDC	AGDR^a^	DRYAD^c^	Zenodo^b^^,^^c^	FigShare
Genome sequence data	+	+	+	+	+	+	+	+	+
WGS annotations	+	?	?	?	?	?	?	?	+
Genotyping data	+	?	?	?	?	?	?	?	+
Transcriptome sequence data	+	+	+	?	?	?	+	+	+
fq.gz	+	+	+	+	+	+	+	+	+
BAM	+	+	+	+	+	+	+	+	+
SFF	+	+	+	+	+	−	+	+	+
HDF	+	+	+	+	+	−	+	+	+
VCF	+	+	+	?	?	?	+	+	+
INSDC-Source	+	+	+	a	b	c	d	*e*	f

The ‘+’ and ‘−’ symbols indicate the presence and absence of the supported data type and data format, respectively. Databases that support any data type beyond the specified most common types are marked by “¥”. Out of the INSDC, source databases were established and maintained by (a) National Genomics Data Centre, China, and China National Center for Bioinformation; (b) The IBDC; (c) New Zealand Ministry for Business Innovation and Employment; (d) University of North Carolina at Chapel Hill, California Digital Library; (e) CERN, the European Organization for Nuclear Research (Conseil européen pour la Recherche nucléaire) and (f) Digital Science. Holtzbricnck Publishing Group, Macmillan Publishers Limited. ‘?’ means that the information is not available. ‘+/−’ means that this data type can be submitted only through a command line or programmatic approach but not by the interactive interface. Detailed information about metadata requirements and database URLs is available in Supplementary Table S1.

a data are available upon request.

b recommended by FAIRsharing.org.

c Databases that support any data type beyond the specified most common types.

Abbreviations: DRA, DDBJ sequence read archive; SFF, Standard Flowgram Format.

**Table 2.** List of Crop/clad Community GGB Databases that integrate various data types, including whole-genome data, genotype, phenotype, QTL, GWAS and germplasm data. Refer to Supplementary Table S3 for data types and metadata for each database

Species/Crop	Database	Database URL
Arabidopsis	TAIR	https://www.arabidopsis.org/
Cassava	CassavaBase	https://www.cassavabase.org/
Citrus	Citrus Genome Database	https://www.citrusgenomedb.org/
Citrus/*Diaphorina citri*/Ca. *Liberibacter asiaticus*	Citrus Greening	https://www.citrusgreening.org/
Cotton	CottonGen	https://www.cottongen.org/
Cucurbit	Cucurbit Genomics	http://cucurbitgenomics.org/
Forest trees	TreeGenes	https://treegenesdb.org
	Hardwood Genomics	http://www.hardwoodgenomics.org/
Grains	GrainGenes	https://wheat.pw.usda.gov
	Gramene	https://www.gramene.org/
	SorghumBase	https://www.sorghumbase.org/
	Triticeae toolbox, T3	https://wheat.triticeaetoolbox.org/
	WheatIS	https://wheatis.org
	KitBase	http://kitbase.ucdavis.edu/
Legumes	KnowPulse	https://knowpulse.usask.ca/
	Legume Information System	https://www.legumeinfo.org/
	PeanutBase	https://peanutbase.org
Pulses	Pulse Crop Database	https://www.pulsedb.org/
	Soybase	https://www.soybase.org/
Maize	MaizeGDB	https://maizegdb.org/
Musa	MusaBase	https://www.musabase.org/
Rosaceae	Genome Database for Rosaceae	https://www.rosaceae.org/
Solanaceae	Sol Genomics	https://solgenomics.net/
Sweet Potato	SweetPotatoBase	https://www.sweetpotatobase.org/
Vaccinium	Genome Database for Vaccinium	https://www.vaccinium.org/
Yam	YamBase	https://www.yambase.org/
Comparative genomics database used by multiple communities		
A comparative genomics database for ∼300 plant species	Phytozome	https://phytozome-next.jgi.doe.gov/
A comparative genomics database hosting 118 genomes from models, crops, fruits, vegetables, etc.	Gramene	https://www.gramene.org/
Others	AgBase	https://agbase.arizona.edu/
	Bio-Analytic Resource	https://bar.utoronto.ca/

Apart from the public databases hosted in the USA and Europe, the Genome Sequence Archive (GSA, https://ngdc.cncb.ac.cn/gsa) in China follows INSDC-compliant data standards (67). The Indian Biological Data Center (IBDC, https://ibdc.rcb.res.in) is also a public repository in India hosting various life science data. For sequencing data, IBDC provides the INSDC-compatible Indian Nucleotide Data Archive (https://inda.rcb.ac.in/home) with data synchronized to NCBI/ENA/DDBJ and the Indian Nucleotide Data Archive-Controlled Access (https://inda.rcb.ac.in/indasecure/home) for private data. In New Zealand, the Aotearoa Genomic Data Repository (AGDR) hosts genomic data, especially for native *taonga* (‘treasure’ in Maori language) species. The presence of these multiple public databases across different countries and regions could be beneficial for the advancement of research in terms of data availability, collaboration and preservation of unique species. However, challenges related to data harmonization, fragmentation and standardization must be addressed to fully harness these resources’ potential for genotype-to-phenotype research.

The recent availability of data submission to cloud storage is also gaining popularity and contributing to the advancement of research focusing on genotype to phenotype. For example, Amazon Web Services (AWS) offers Open Data (https://aws.amazon.com/opendata) source for unregistered users to find and use publicly available datasets, while allowing subscribed customers to search and access even third-party data (https://docs.aws.amazon.com/data-exchange/index.html) for research use. In addition, through Amazon Omics (https://aws.amazon.com/omics/), it provides data on Plant and Animal Genomics (https://aws.amazon.com/solutions/agriculture/plant-animal-genomics/), which is another platform that could be used to facilitate omics data analysis and integration. Similarly, there are other cloud storage options for storing and accessing genetic data for research by users, either corporate and individuals, including Google Cloud Life Sciences (https://cloud.google.com/life-sciences) and Microsoft Genomics (https://azure.microsoft.com/en-in/products/genomics/).

Table 1 contains a list of active, maintained and updated public repositories for genome, genotyping and transcriptome sequence data. Detailed information about metadata file formats related to these repositories is provided in Supplementary Table S1.

The metadata associated with sequence and genotype data promotes a dataset’s discoverability and reusability. We note here that many secondary public repositories exist that exclusively host data on promoters, transcription factors, proteomes, various RNA types, epigenomics data and pangenomes (Supplementary Table S2). However, here, we limit our discussion to primary genotype and phenotype data and expect that detailed discussions on other related topics will be provided by the other working groups of the AgBiodata consortium.

#### Metadata requirements on genomics and transcriptomics datasets

The metadata associated with genome, genotyping and transcriptome sequencing is crucial for data reusability and interoperability. To maximize the implementation of FAIR standards, the metadata should be described with accurate Gene Ontology and Plant Ontology terms with proper evidence codes wherever applicable. Project- and sample-level metadata typically includes taxonomic identifier (for species), tissue type (organism part) from which the sample was taken, disease state, growth or developmental stage of the sample, the biological gender of the sample and collection date. Assay-level metadata is directly related to the preparation of biological materials undergoing the assay, including method details (bulk RNA-seq, scRNA-seq, etc.), library information (single-end or paired-end), replicates (biological or technical), instrument metadata, quality control (QC) and workflow metadata. For example, submission of sequencing data to NCBI GenBank and SRA requires metadata for the submitter (including name, affiliation, and email of the data submitter and other authors), BioProject goals (such as genome sequencing and assembly; raw sequence reads, epigenomics, exome, proteome and variation) and BioSamples information (like organism’s name and taxonomic identifier, geographical origin of the sample and tissue type).

We note here that in most repositories, the organism’s name is the only required field for biological targets, with optional fields of strain, breed, cultivar, isolate name, label and description. The minimum general information required for a project is the data release date, project title and public description of the study goals. Optional fields include a project’s relevance to a field (agricultural, medical, industrial, environmental, evolution, model organism and other), external links to other websites associated with the study, grant information (number, title and grantee), research consortium name and the uniform resource locator (URL), data provider and URL (if different from the submitting organization) and publication information.

Optional but useful metadata for BioSamples includes sample title, BioProject accession, biomaterial provider (laboratory name and address, or a cultural collection identifier), name of the cell line, cell type, collected by and date, culture identifier and source institute (refer to http://www.insdc.org/controlled-vocabulary-culturecollection-qualifier), disease name and stage, observed genotype, growth protocol, height or length measured, the growth environmental, the geographical coordinates of the sample collection, phenotype of sampled organism (compliant with the BioPortal at http://bioportal.bioontology.org), population (filial generation, number of progeny and genetic structure), sample type (cell culture, mixed culture, tissue sample, whole organism, single cell and so on), sex, specimen voucher, temperature of the sample at time of sampling, treatment and sample description (defined in the Phenotype And Trait Ontology of Open Biological and Biomedical Ontology Foundry at http://obofoundry.org/ontology/pato.html).

The mandatory attributes for library construction metadata are BioSample name, library ID, a title, data type and method information (Whole Genome Amplification, Whole Genome Sequencing (WGS), RNA-Seq, Expressed Sequence Tags, ChIP-Seq, and so on), source (GENOMIC, TRANSCRIPTOMIC, GENOMIC SINGLE CELL, METAGENOMIC, etc.), selection (Polymerase Chain Reaction (PCR), RANDOM, Reverse Transcription-PCR, cDNA, DNAse, Restriction Digest, etc.), layout, platform, instrument model, design description, file type and filename(s).

A few other sequence data repositories do not enforce the submission of metadata but encourage data submitters to provide as many details as possible. In this category, AGDR (https://repo.data.nesi.org.nz/DD) requires submitter ID, project ID, project code, project name, program name, database gap accession number, experiment type, number of samples and replicates and data type. In addition, it provides metadata templates for submitting detailed information on samples and methods (sample, aliquot, RNA integrity number-, adapter name and sequence, barcoding, base caller name and version, experiment name, flowcell barcode, fragment sizes, instrument model, lane number, library name and library preparation kits), project, publication, core metadata collection, indigenous governance and indigenous knowledge label templates.

The minimum metadata for a DRYAD submission requires a title describing the data and the study, author(s) information, abstract (dataset structure and concepts, reuse potential, legal or ethical considerations, etc.) and research domain. Optional metadata recommended is funding information, research facility, keywords, technical methods details and publication details. However, the biosample or plant accession metadata is not captured. Figshare recommends metadata submission guidelines similar to INSDC repositories but does not enforce them as a requirement. The storage quota for a free account is 20 GB and up to 100 projects.

#### Genotyping data submission and metadata requirements

The major repository for submitting non-human VCF files containing genotyping-related data is the European Molecular Biology Laboratory-European Bioinformatics Institute (EMBL-EBI) European Variation Archive (EVA) (68), but a newer repository has also arisen in the Genome Variation Map (69). NCBI hosts the dbSNP and dbVar databases, which are intended for human data. All repositories strive to adhere to FAIR practices, but others have put forth additional recommendations (70). The EVA repository accepts VCF file structures, including hapmap formatted files (71) and SNP genotyping arrays, that are validated using custom EBI VCF Validation Suite software (https://github.com/EBIvariation/vcf-validator) with a minimum number of data fields with accompanying metadata that includes, but is not limited to, project title, sequencing platform information, software, reference organism and genome version and date and data generation location. The data fields for a VCF are the header lines that contain information about the dataset and relevant reference sources (organism, genome version, alignment, mapping method, etc.) followed by the variant site record row data: chromosome number, chromosome position, reference allele, alternate allele, quality, filter tag and additional allele info format (https://gatk.broadinstitute.org). However, the naming structure within some of these fields is not standardized, which can lead to interoperability concerns.

#### Crop Community GGB Databases

Whole-genome, transcriptome and genotype data can also be submitted to most of the GGB databases such as Genome Database for Rosaceae (72, 73), CottonGen (74, 75), SoyBase (76, 77), Legume Information System (78, 79), Sol Genomics Network (80, 81), MaizeGDB (82, 83), TreeGenes (84, 85), the Arabidopsis Information Resource (TAIR) (86, 87), KnowPulse (88) and InterMine (89, 90) (Table 2). Some of these databases, such as Gramene (91–93), SorghumBase (94) and InterMine (89, 95), do not accept data from authors but obtain from the primary databases. Depending on the GGB databases, different types of data and metadata can be submitted. Typically, these crop GGB databases collect a wide variety of data such as quantitative trait loci (QTL), GWAS, markers, alleles, genetic maps and cultivar/germplasm phenotype data and integrate them with whole-genome, transcriptome and genotyping data. These GGB databases standardize various names that associate the data with various ontologies to integrate data from various sources and of various types. This integration of different kinds of data, not typically done in the primary databases specialized in particular types of data, is one of the key steps in making the data FAIR. Integrating data from diverse sources provides researchers with foundations for subsequent statistical analyses to discover novel associations between different data types, potentially leading to valuable insights and breakthroughs. For example, SNP genotype data and phenotype data from multiple locations of the same germplasm allow further analysis that can reveal how particular genotypes manifest specific phenotypes in distinct environments.

#### Uses and Applications

WGS data can be reused in genome assembly (96–98); pan-genome construction (99); single-nucleotide variation (100–102), copy number variation (CNV) (103–105) and structure variation (SV) (106–108) discovery; phylogenomics; comparative genomics and other genome research to study genome structures, genome diversity, the evolution of gene families or organisms, crop domestication and improvement (69, 109–111). Genotyping data in VCF format (https://samtools.github.io/hts-specs/VCFv4.2.pdf) can be used for numerous purposes: to store the location of given variants (including GWAS-associated variants), to identify targets of molecular markers for genotyping purposes, to evaluate the effects of given base pair and structural variants on gene function, to perform comparative genomics and evolutionary studies and to assist computational breeding approaches via machine learning and other methods. Data extraction and manipulation of VCF files are easy with the use of existing software toolkits such as VCFtools (51) (https://vcftools.github.io/index.html) and SAMtools (112) and can be utilized in conjunction with existing and ad hoc bioinformatic pipelines due to their command line functionality. By integrating VCF data with RNA-Seq and phenomics data, researchers can use these data sets for quantitative genetic studies, including GWASs (113–116), QTL (115, 117–119) analysis, marker discovery and genome selection (GS) (120–122) to accelerate modern breeding techniques. Integrating transcriptomics data with metabolomics data can help predict biomarkers often associated with biological pathways. This will assist in understanding the mechanism of underlying molecular patterns driving a condition. Integration of genomic, epigenomic and transcriptomic profiles will facilitate the prediction of key genomic variables and biological variation. Integration of gene expression data and CNVs can be used to categorize samples into groups based on their similarity to two datasets.

## Phenotype and Phenomics

### Data types, Repositories and Knowledge Bases

Plant phenotyping is the key for plant breeding, characterization of biodiversity and genetic and genomic-based approaches for translational research (123). The classical genetic and functional genomics studies in model and crop plants have identified numerous mutants that show distinct morphological and anatomical phenotypes associated with one or more genes, pathways and molecular processes. Table 3 lists databases that host the mutant collections and description of the phenotype of individual mutants and associated genes, including MaizeDIG (83), RIKEN Arabidopsis Genome Encyclopedia (124), Mutant Variety Database (125), Plant Genome Editing Database (126), Tomato Mutants Archive TOMATOMA (127) and Plant Editosome Database (109).

**Table 3.** List of public repositories, databases and secondary knowledgebases hosting or integrating various types of phenotypes, phenomics and molecular phenotype data

Category	Databases	URLs
Species-specific mutant collections	Database of images and genome (MaizeDIG)	https://maizedig.maizegdb.org/
	Mutant Variety Database	https://nucleus.iaea.org/sites/mvd/SitePages/Home.aspx
	Plant Genome Editing Database	http://plantcrispr.org/cgi-bin/crispr/index.cgi
	RIKEN Arabidopsis Genome Encyclopedia	http://rarge-v2.psc.riken.jp/line
	TOMATOMA	https://tomatoma.nbrp.jp/index.jsp
	Plant Editosome	https://ngdc.cncb.ac.cn/ped/
Traits and QTLs	Gramene QTL	https://archive.gramene.org/qtl/
	Wheatqtl	http://www.wheatqtldb.net/
	GLOPNET	http://bio.mq.edu.au/∼iwright/glopian.htm
	TRY database	https://www.try-db.org/TryWeb/Home.php
	Ecological Flora of the Britain and Ireland	http://ecoflora.org.uk/
	BIOPOP	http://www.landeco.uni-oldenburg.de/Projects/biopop/main.htm
	FloraWeb	https://www.floraweb.de/
	USDA GRIN	https://www.ars-grin.gov/
	BiolFlor	https://wiki.ufz.de/biolflor/index.jsp
	LEDA	https://uol.de/en/landeco/research/leda
	USDA PLANTS	https://plants.usda.gov/home
	BROT	https://www.uv.es/jgpausas/brot.htm
	AusTraits	https://austraits.org/
	Community Databases in Table 2 and Supplementary Table S3	
Phenomics	GnpIS	https://urgi.versailles.inra.fr/gnpis
	PGP Repository	https://edal-pgp.ipk-gatersleben.de/
	Cartograplant	https://cartograplant.org/
	AgData commons Plants & Crops	https://data.nal.usda.gov/ag-data-commons-hierarchy/plants-crops
	PathoPlant	http://www.pathoplant.de/
	PncStress	http://bis.zju.edu.cn/pncstress/
	Indian Crop Phenome DB	https://ibdc.rcb.res.in/icpd/
Gene Expression	Ozone Stress Responsive Gene Database	https://www.osrgd.com
	EBI-Plant Expression Atlas	https://www.ebi.ac.uk/gxa/plant/experiments
	CoNeKT	https://conekt.sbs.ntu.edu.sg/
Protein, peptides and proteomes	Expath	http://expath.itps.ncku.edu.tw/
	Proteome Xchange	https://wwwz.proteomexchange.org
	Plant Proteome Database	http://ppdb.tc.cornell.edu/
	PlantMWpIDB	https://plantmwpidb.com/
	Heat Shock Proteins database	http://hsfdb.bio2db.com/
	WallProtDB	https://www.polebio.lrsv.ups-tlse.fr/WallProtDB/
	Aramemnon	http://aramemnon.botanik.uni-koeln.de/
	PhosPhAt	https://phosphat.uni-hohenheim.de/db.html
	Database of Phospho-sites in Plants	http://dbppt.biocuckoo.org/browse.php
	Plant Protein Phosphorylation Database	https://www.p3db.org/home
	qPTMplants	http://qptmplants.omicsbio.info/
	Plant PTM viewer	https://www.psb.ugent.be/webtools/ptm-viewer/
	PlaPPISite	http://zzdlab.com/plappisite/index.ph
	*M. truncatula* Small Secreted Peptide Database	https://mtsspdb.zhaolab.org/database
	PlantPepDB	http://14.139.61.8/PlantPepDB/index.php
	Arabidopsis PeptideAtlas	http://www.peptideatlas.org/builds/arabidopsis/
	Indian Structural Data Archive	https://isda.rcb.ac.in/
Metabolites, biochemical and small chemical entities	Antimicrobial plant peptides (PhytAMP)	http://phytamp.pfba-lab-tun.org/main.php
	PubChem	https://pubchem.ncbi.nlm.nih.gov
	ChEBI	https://www.ebi.ac.uk/chebi
	Metabolomics Workbench	https://www.metabolomicsworkbench.org
Secondary Knowledgebase	MetaboLights	https://www.ebi.ac.uk/metabolights/index
	PoDP	https://pairedomicsdata.bioinformatics.nl/
	Plant Reactome pathway knowledgebase	https://plantreactome.gramene.org
	MetaCyc	https://metacyc.org
	PMN	https://plantcyc.org/data
	KEGG pathways	https://www.genome.jp/kegg/pathway.html
	PlantPathMarks (PPMdb)	http://ppmdb.easyomics.org/
	The Bio-Analytic Resource	https://bar.utoronto.ca
	The protein–protein interaction database for Maize	https://mai.fudan.edu.cn/ppim/

Abbreviations: PTM, Post-translational modifications; PMN, Plant metabolic network; KEGG, Kyoto encyclopedia of genes and genomes.

In addition, complex phenotypic traits (i.e. morphological and physiological) related to the fitness and performance of an organism are often quantitative and have multiple genetic determinants (128, 129). Examples of traits determined by multiple genes (known as QTLs) are crop yield, biomass, resistance to pests and pathogens, abiotic stress tolerance, nutritional value and ease of harvest. In addition to crop breeding, trait-based approaches are widespread in ecological research (130), as they provide a general understanding of a wide range of ecological and evolutionary phenomena, such as the impact of climate change and anthropogenic land use on biodiversity (131–133). In Table 3, we provide a list of key databases (or portal of bigger databases) that host information related to traits, QTLs and associated data including the Gramene QTL database (134), QTL database for wheat (135), Global Plant Trait Network Database (GLOPNET) (136), TRY Plant Trait Database (137), a database of Ecological Flora of the Britain and Ireland (138), BIOPOP Database of Plant Traits (139), GRIN (140), the United States Department of Agriculture (USDA) PLANTS Database, BiolFlor (141), LEDA Traitbase (142), BROT database of plant traits for Mediterranean basin species (143) and AusTraits (144). Trait and QTL data are also integrated with other types of data in various crop community databases listed in Table 2.

Phenomics is the systematic analysis for the refinement and characterization of phenotypes on a genome-wide scale. With the advent of high-throughput platforms, it became possible to collect phenomics data at a single-cell, organismal and/or population-wide scale (145). Phenomics can be used for species recognition and biodiversity characterization (146), stress quantification (146–148) and crop yield prediction (149, 150). Thus, phenomics datasets are very large and have different formats (e.g. JavaScript Object Notation files). Some of the databases that host phenomics data include Genoplante Information System (GnpIS) (151, 152), Plant Genomics & Phenomics Research Data Repository (PGP) (153), Cartograplant (154), AgData commons (https://data.nal.usda.gov/ (155), PathoPlant (156, 157), PncStress (158) and Ozone Stress Responsive Gene Database (159).

Despite its analogy to genomes, it is not possible to fully characterize phenomes due to heterogeneity and multifaceted nature of phenotype data with added layers reflecting complexities at the cell, tissue and whole plant levels that have further variations according to the development stages and growth environment (145, 160). Thus, phenomics approaches may focus on specific factors of phenotypic data. For example, an intensive phenomics study may focus on high-throughput digital imaging across different stages and tissues of an organism in different growth stages or growth environments and may include quantitative data about plant height, biomass, flowering time, yield and photosynthesis efficiency. Another study may employ orthomosaic or time series Red, Green, Blue images and remote sensing to monitor the algal blooms in the ocean (161) or lesions in maize leaves (162). As phenomics data can be highly variable, necessary metadata includes information about plant species, tissue, developmental stage, environmental conditions, experimental design and data collection, processing, and analysis.

In addition to traditional phenotypes, molecular phenotypes include changes in the chromatin organization, transcripts, proteins, metabolites and ions (163–165). The quantitative changes in the gene expression, proteins and metabolite profiles in plants have far-reaching consequences for (i) the nutritional values of cereals, legumes, fruits and vegetables; (ii) the quality of bioproducts such as wine, beverages, vinegar, oil and fuel; (iii) the ability of plants to adapt in response to various abiotic stress conditions and (iv) the innate ability to defend against pests, pathogens and herbivores (166–170).

Proteome and metabolome datasets allow a deeper understanding of an organism’s metabolic processes at the level of organ, tissue and cell, as well as how these processes change in response to intrinsic developmental programs and environmental factors. Proteome datasets further confirm the subcellular localization, their comparative abundance between different tissues and cells, protein–protein interactions and post-translational modifications (171). Once the original proteomic datasets and associated metadata/manuscript have been submitted to public data repositories such as Proteomics Identifications database (171–173), MassIVE (https://massive.ucsd.edu/ProteoSAFe/static/massive.jsp), Japan Proteome Standard Database (174, 175), Integrated Proteome resources (176, 177), Panorama (178) and Peptide Atlas (179, 180), they are made available for re-analysis and further exploration by other researchers. Metabolomics provides a comprehensive overview of the metabolite profile of an organism, tissues, cells or subcellular component at a specific time point and is used to identify nutritional, medicinal, flavor and disease resistance compounds as well as chemical interactions between plants and other biological systems. A recent comprehensive review of the methodologies to explore the highly complex and diverse metabolites of plants and associated methodologies can be found in (181). The types of data collected for metabolomics depend on the method of chemical fingerprinting. As an example, in mass spectrometry (MS), a typical dataset would consist of a matrix containing information on the retention time and index, the mass-to-charge ratio (*m*/*z*) and peak characteristics such as the number and width. These data go through pre-processing, which converts raw instrument data into organized formats using background subtraction, noise reduction, curve resolution, peak picking, peak thresholding and spectral deconvolution. There are various software tools for analyzing metabolite data, each of which may be specific to a particular method of detection or instrument used in the analysis. The most popular software packages are MZmine, XCMS, MSdial, metaMS, Progenesis QI and MetAlign. For annotation of unknown metabolites, popular software tools include MS-FINDER, MetDNA, MetFamily and GNPS, among others. Raw file formats generated by the machines included raw, idb, cdf, wiff, scan, dat, cmp, cdf.cmp, lcd, abf, jpf, xps and mgf. Derived file formats are mzml, nmrml, mzxml, xml, mzdata, cef, cnx, peakml, xy, smp and scan. Several initiatives were undertaken due to the complexity of metabolomic data. The Chemical Analyses Working group started the **‘**Metabolomics Standard Initiative’ to develop metabolomic standards (182, 183) with revisions suggested by (184). Community-driven Metabolomics Society has a Data Standards Task Group focusing on metabolomics data standardization and sharing. This was followed by the ‘Coordination of Standards in Metabolomics’ (185), and MetaboLights (186), for developing tools to ease the submission of metabolomic data (187). ProteomeCentral and Omics DI serve as central repositories for these datasets, which are then reused in protein knowledge bases (UniProt and NeXtProt), genome browsers (Ensembl and University of California Santa Cruz), proteomics resources and other bioinformatics resources (e.g. OpenProt and LNCipedia). The ProteomeXchange (PX) datasets are re-analyzed by different proteomics resources of the PX consortium, making data more reliable. The Paired Omics Data Platform (PoDP) (188) links the metabolomics data submitted to MassIVE or MetaboLights to genomes stored in NCBI or JGI. In Table 3, we list the two major repositories available for submission of raw and processed metabolome data, the National Institutes of Health Common Fund’s National Metabolomics Data Repository portal and the Metabolomics Workbench and MetaboLights.

Some gene expression and metabolic phenotypes often culminate in visible phenotypes, which can be described using the Plant Ontology terms (189–191). More recently, Plant Ontology terms have been extended to large-scale phenomics data from a single species (192) to support the comparative phenomics in plants (193) and describe trait phenotypes expressed under a specific developmental stage or specific environment and stress (194). To cover the genotype–phenotype gap, we need to integrate multiple types of data, including genotypic, large-scale phenome, gene expression, proteome and metabolome data, described using defined and standardized ontologies.

Finally, making phenotype data FAIR requires developing additional public repositories and community guidelines for standardization and formatting phenotype data with well-described metadata. Furthermore, new tools and features are in development for the visualization of phenotype data on genome browser (195). The phenotype data and derived information can also be integrated into plant metabolic networks (196, 197), system-level plant pathways (198–200), expression Atlas, metabolic models and so on. The integration of genotype and phenotype information in the secondary knowledge bases is of primary importance to plant researchers for formulating data-driven hypotheses as well as for analyzing the high-throughput omics data (196). Here, we provide a list of public repositories and knowledgebases currently hosting various types of phenotype data in Table 3.

### Phenotype data formats, standards and metadata

The structure and characteristics of data types and any additional metadata are crucial for enabling future data reuse and re-analysis by other researchers. The most relevant metadata shared across the various data types (generated by a diverse set of methods and platforms) includes taxonomic identification of the plant, the individual or cultivar name or accession ID, georeferences or growth conditions, field sampling or experimental design, cell, tissue, organ information (e.g. whole plant, leaf, root, flower, shoot, single cell, etc.), plant maturity and health status, measurement date (season, time of the day) and the type of phenotype measured (quantitative or qualitative) (137, 201). These metadata can be entered as a simple text format during the submission of the raw data to any primary repository and are easily exported from one database to another as text files.

Furthermore, plant phenotypes can be classified as categorical (qualitative and ordinal) or quantitative (continuous) traits (15). Some phenotypes are rather stable within species (mostly categorical traits), and some of these can be systematically compiled from species checklists and floras (202). Thus, not all phenotypes can be mapped from one species to another. It is also important to note here that often, a phenotype is a cumulative outcome of the genotype, the environment and their interaction. Many important agronomic traits, such as seed or fruit quality, yield, abiotic stress tolerance and pathogen resistance, have a quantitative genetic architecture involving minor and major genes or QTLs. Thus, the research question and the method become important to set the scope and goals of the study and require specific metadata and standards. For instance, most traits relevant to ecology and earth system sciences are characterized by intraspecific variability and trait–environment relationships (mostly quantitative traits). These traits must be measured on individual plants in their particular environmental context. Each such trait measurement has high information content as it captures the specific response of a given genome to the prevailing environmental conditions (137). Thus, the collection of these quantitative phenotype data and their essential environmental covariates is of vital importance. While trait measurements themselves may be relatively simple, the selection of the adequate entity (e.g. a representative plant in a community or a representative leaf on a tree) and obtaining the relevant ancillary data (taxonomic identification, soil and climate properties, disturbance history, etc.) may require sophisticated instruments and a high degree of expertise and experience. Besides, these data are most often individual measurements with a low degree of automation. This limits the number of measurements and causes a high risk of errors, which need to be corrected *a posteriori*, requiring substantial human work. Hence, the integration of these data from different sources into a consistent dataset requires a carefully designed workflow with sufficient data quality assurance. These measurements of quantitative traits are single sampling events for particular individuals at certain locations and times, which preserve relevant information on intraspecific variation and provide the necessary detail to address questions at the level of populations or communities (201). Hence, an accurate and careful collection of data, including their associated metadata and ancillary data, is key to correctly preserving this valuable information and performing a suitable data integration across studies, species and data types.

## GWAS and QTL mapping

GWAS (203–206) and QTL (207–209) mapping are statistical methods used to identify marker-trait associations and candidate genes (causative mutations) controlling traits of interest (210). Both approaches rely on the linkage disequilibrium between the tested markers and the functional polymorphisms at the causative genes (211, 212). However, they differ on the type of genetic populations used for the study (204, 213): GWAS relies on a diversity panel (*e.g.* germplasm collections) of, ideally, unrelated individuals; on the contrary, QTL mapping investigates the co-segregation of genetic markers with desired phenotypes in progeny purposely generated (e.g. F_2_ population or recombinant inbred lines) (210). Although both these analytical methods, their results can be used as data inputs for other types of analysis (e.g. meta-analysis, estimation of polygenic scores) (214). The genomic and genetic positions of trait-associated markers from GWAS and QTL studies can also be integrated with other types of data, enabling data transfer among related species. Thus, their outputs can be considered a data type, and consequently, they require metadata collection and the use of standards in order to make them FAIR. Therefore, the FAIRness of the association mapping outputs is vital in linking genotype and phenotype in the multi-omics era.

The primary output of a GWAS analysis is a list of variant positions, SNP ID or indel positions, allele, strand information, effect size and associated standard error, *P*-value and corrected *p*-value, test statistics, minor allele frequency and sample size (215). One critical metadata for GWAS/QTL data is the statistical method used to calculate and correct the *P*-values (GWAS/QTL). Regarding the SNPs, the most important metadata includes the model species and the version of the reference genome against which these SNPs are mapped (refer to the Genotype data section). The metadata required to make the traits interoperable and reusable is explained in the laboratory/field traits section. In the case of QTL analysis, a linkage map and pedigree information of the individuals, as well as the heritability of each SNP, is also important to be collected (115).

Unlike the human and animal GWAS and QTL data, open access resources such as the National Human Genome Research Institute-EBI GWAS Catalog, GWAS Atlas (216), OpenGWAS, Animal QTL database, and Animal Genome Informatics resources (USDA national infrastructure National Research Support Project: A National Animal Genome Research Program), QTL and GWAS data for plant species and major crops are mostly stored in crop community database (Table 2). The databases typically integrate the QTL and GWAS data with other types of data, playing a crucial role in improving the findability and accessibility of plant GWAS data that would have otherwise been buried in publications. AraGWAS Catalog (216) contains recomputed GWAS results using a standardized GWAS pipeline on all publicly available phenotypes from AraPheno (217).

Meta-analysis is a widely used analysis for integrating the summary statistics from multiple GWAS/QTL studies (218, 219). It is a set of methods that allows the quantitative combination of data from numerous studies and the evaluation of the consistency, inconsistency or heterogeneity of the results across multiple datasets (218). Meta-analysis of GWAS/QTL datasets can improve the power to detect association signals by increasing sample size and examining more variants throughout the genome than each dataset alone (220). However, in order to integrate datasets coming from different studies in meta-analysis, standardized data and metadata collection across the studies are needed (16). In addition, the genotype and phenotype data from the GWAS/QTL studies can be reused for further knowledge discovery, especially for QTL by environment interaction, predicting plant response in new environments and linking genomes to complex phenotypes across species (221, 222).

## Data reusability limitations and challenges

Accessing, reusing and integrating analytic data from various data types remain difficult (223). Despite the significant progress made in agricultural research due to advances in genotyping and phenotyping technologies, most of the data used and generated in research studies are not shared. Here, we discuss limitations to data reuse in genotype-to-phenotype studies in three aspects: challenges with data, resources and funding and implementation of FAIR data policy.

### Challenges with data

#### Data diversity and data format heterogeneity

Agriculture and horticulture research involves a wide range of genotypic, phenotypic and environmental data, often from different experimental protocols, data generation technologies and data processing workflows. As a result, data formats can be highly heterogeneous, making it challenging to integrate data from different sources and reuse them in future studies (137). This issue is even more significant for phenotypic data, especially with emerging high-throughput phenotyping technologies. Digital imaging and remote sensing allow researchers to explore new levels of trait variability that were previously inaccessible using traditional and manual phenotyping methods. However, the large diversity of data and metadata generated by these technologies can be highly variable in terms of file size, format and content. The heterogeneity of the data analysis pipeline also contributes to the complexity of standardization in phenomics.

#### Data size, quality and versioning

Most genomics, transcriptomics, epigenetics and phenomics data are extremely large in file size and computationally intensive. For example, whole-genome sequencing data used for variant calling or VCF files that collate multi-individual genome-wide variants can be computationally challenging to handle, limiting their sharing in FAIR public repositories and making data manipulation difficult. Also, data quality and integrity may be compromised before or during the submission process, preventing reuse.

#### Object identification

Data submitted to a public domain often lack a unique data object identifier (DOI) and any plant or accession identifier (PID), which makes it challenging to trace and integrate different types of data generated from the same individual plant across experiments and research laboratories. Having a universal DOI associated with its PID would be desirable to improve data findability and reuse. However, most data used and generated in research studies are not shared, inaccessible or reusable because of missing fundamental metadata or improper data format.

#### Metadata and data standardization

Metadata is any type of data descriptor that can facilitate data interpretation and reuse. It is very common that when data are submitted to public domains they are accompanied by incomplete, inconsistent or missing metadata. Developing and promoting standard data formats and metadata can improve data discovery and reuse, facilitate data integration and interoperability and allow data from different sources. Some data standards for genomics and phenomics data have been developed, such as the Minimum Information About a Genome Sequence from the Genomic Data Standards Consortium, the Plant Phenotype Ontology and the Minimum Information About a Plant Phenotyping Experiment. For GWAS data, GWAS-VCF format has been proposed. However, promoting and consistently applying these standards across different research groups and databases remain challenging. For instance, if there are standards for collecting and describing trait measurements, they are organism-specific (e.g. International Organization of Vine and Wine; www.oiv.int) or based on model species.

The metabolomic research community faces similar challenges. The USA Plant, Algae, and Microbial Metabolomics Research Coordination Network (224) coordinated an initiative to identify the grand challenges of metabolomic research. As noted, the data obtained from metabolomic analyses can often result in different chemical feature values even in the same biological treatments due to the variability associated with biological systems, equipment differences and protocol and reagents. Therefore, identifying metabolites with confidence and the limited metabolome depth of coverage are the key challenges in metabolomic research (225). A recent review of liquid chromatography-mass spectrometry (LC-MS) literature found a lack of details reported on the methodology and level of confidence for metabolites in most of the reviewed research articles (226). To address these challenges, multi-dimensional analysis methods, the use of standard libraries for metabolite characterization and tools that simplify the submission of metadata and data are being developed (187, 227).

### Resources and funding

The submission of different data types (i.e. genomics, transcriptomics, proteomics, metabolomics and phenomics data) to separate and specialized primary repositories is a common practice, resulting in a heterogeneity of data repositories and multiple PIDs, limiting data interoperability. It is challenging to locate phenotypic datasets for a particular set of plants that have been characterized at the genomic or transcriptomic level due to the absence of common standards among data repositories.

Incompatible software or hardware among different data platforms also makes interoperability challenging. Bulk data download or data movement across repositories is another issue due to data size and a lack of standardization. Software development and maintenance are required for fast data search and retrieval and sufficient user support. For some types of plant data, such as QTL and GWAS, there are no primary repositories where researchers can submit their data. Community GGB databases (Table 2) address this issue by collecting, curating and integrating various data types from different sources and related species, which is pivotal in data integration. However, not all plant GWAS data are timely stored in databases due to either a lack of crop community databases or funding for curation. In addition, community GGB databases often have limited computational and personnel resources for curation and inclusion of all types of omics data due to limited funding and lack of understanding of the importance of curation by funders. Additionally, there is a lack of appropriate infrastructure for the raw data deposition in community databases.

### Implementation of FAIR data policy

FAIR data policy refers to the list of 15 guidelines elaborated to facilitate data search, access and reuse by human-driven and machine-driven activities (228). These principles apply to every type of scholarly digital object archived in a repository, and their implementation has started in many different research fields (224, 229, 230). In summary, these principles recommend that, when data are submitted, they are very well described using detailed metadata and are assigned a globally unique and persistent identifier that allows everybody to find them in a searchable resource. Data should be formatted according to community-based standards if available or in a way that human and computer systems can easily interpret and exchange. Controlled vocabularies and ontologies are strongly encouraged to facilitate data interoperability across database resources. FAIR data, however, do not mean open access or free but refer to clarity and transparency about the conditions governing access and reuse (e.g. credential system to access and download data (231). These principles aim to increase data transparency and improve data reuse for new research purposes, enhancing data value over time.

The implementation of the FAIR data policy, however, can be challenging due to several reasons. First, making data FAIR requires additional efforts and time commitment from researchers for which adequate training and funding may not exist. Second, many scientists are unaware of the FAIR principles, community-based standards, ontologies and public databases. Third, there is also a need for long-term sustainability of databases and bioinformatic training/outreach, which requires ongoing funding and infrastructure support. Many databases struggle to secure funding and may face difficulties in maintaining FAIR data and providing training to its users. Other barriers for FAIR data include considerations of data privacy and confidentiality, legal and ethical issues, concerns of ownership and lack of incentive if not credited for sharing data.

## Recommendations

Big data generated by recent high-throughput sequencing and phenotyping technologies allow researchers to use datasets to explore how an organism’s genetic code influences its physical traits. To accelerate G2P research, we propose the following recommendations for ensuring data interoperability and reuse for discovering new knowledge and promoting translational research.

- Standardization of data collection protocols: Standardizing data collection protocols and using common data formats are recommended to be developed by each crop community to ensure that data are collected consistently and comparably. Using metadata standards will make sharing and comparing data across different studies easier.
- Centralized or interoperable data sharing platform: It is beneficial to have a centralized data sharing platform, but it is also recognized that multiple database resources can be built with different strengths. It is recommended for these resources to use standardized data models and exchange formats and the deployment of existing and emerging software components to facilitate the sharing of genotypic and phenotypic data. It includes the use of online databases and repositories that are specifically designed for the storage and sharing of plant genetic and phenotypic data.
- Consistent data annotation: It is recommended that researchers consistently annotate data with relevant information such as the genotype, phenotype and experimental treatments to make the data more easily searchable and usable by other researchers.
- Data QC: It is recommended that researchers use more automated management of data flows and implement data QC such as data curation and validation to ensure that the data are accurate, are reliable and can be used to make valid conclusions.
- Data integration: It is recommended that databases adopt new database technologies and develop robust data standards that can facilitate the global integration of G2P data in the future. Data integration from different resources such as genomics, transcriptomics, proteomics and metabolomics can help to better understand the complex relationship between the genotype and the phenotype.
- Community-driven efforts: It is recommended that researchers and funders make more community-driven efforts such as open-source projects, workshops and collaborations that can help to promote the sharing and use of data among researchers, which in turn will lead to a better understanding of the G2P relationship. There should be encouragement for integrated science training plans that enable biologists to think quantitatively and facilitate collaboration with experts in physical, computational and engineering sciences. It can help scientists get familiar with the development of computational pipelines and workflows that will be essential for researchers to acquire, analyze and critically interpret G2P data.
- Data storage infrastructure, data management software and data curation tools: Funders are recommended to recognize that these tools are necessary to handle large volumes of data in diverse formats and have researchers to have a separate funding for this type of work, ideally in collaboration with the existing community databases instead of reinventing the wheel.
- A concerted effort to make multi-omics datasets interoperable by biocuration with controlled ontology terms will help address this issue. Community databases address some of these issues by collecting, curating and integrating various data of different types from different sources and from different but related species. However, community databases need to have sustainable funding.
- Data security, backup and recovery must be considered and implemented for sustainability.
- Data compliance with data sharing policies, privacy regulations and laws should be enforced.

## Supplementary Material

baad088_Supp
