# Supplementary material for: Genotype and phenotype data standardization, utilization and integration in the big data era for agricultural sciences
Source: Database (Oxford). 2023 Dec 11;2023:baad088. doi: 10.1093/database/baad088 (PMC10712715; doi:10.1093/database/baad088)
Supplement: baad088_Supp [file baad088_supp.zip › suppl_data/SuppTable1.docx]

| **Supplementary Table 1. Metadata description.** | | |  |
| --- | --- | --- | --- |
| **Database** | **Description** | **Link for metadata** | **Note** |
| **NCBI** | NCBI provides BioSample metadata templates.based on organism linearage validation | (https://www.ncbi.nlm.nih.gov/biosample/docs/submission/validation/) | INSDC standard metadata is required for project, sample, and sequence library |
| **DRA** | DRA explains metadata requirements in detail for six object types: Data Submission, BioProject, BioSample, Experiment, Run, and Analysis. | https://www.ddbj.nig.ac.jp/dra/metadata-e.html | INSDC standard |
| **ENA** | ENA provides Sample checklists including Genomic Standards Consortium (GSC) package extension for reporting plant measurements and observations | https://www.ebi.ac.uk/ena/browser/view/ERC000020 | INSDC standard |
| **GSA** | GSA is INSDC compatible. Metadata is required for BioProject, BioSample, Experiment, and Run. | https://ngdc.cncb.ac.cn/gsa/document/start_here.jsp#handbook | GSA provides tutorials, detailed descriptions, and quickstart guides about metadata in Chinese and English. |
| **AGDR** | Metadata are defined for administrative entities (program, project, experiment, publication, indigenous governance, etc.), Biospecimen, BioSample, and data file. | https://repo.data.nesi.org.nz/DD | The AGDR follows the principles of Māori Data Sovereignty. Metadata templates can be downloaded as Json or TSV files. |
| **DRYAD** | Minimum metadata required includes journal name, title of the study, authors, abstract about the dataset structure and concepts, and research domain. | https://datadryad.org/stash/submission_process#upload-methods | It is suggested file sizes not exceed 10GB for efficient upload/download, with a maximum of 300GB per data submission made through the web interface. |
| **Zenodo** | Metadata is compliant with the minimum and recommended terms in DataCite's Metadata Schema. Data is versioned and a DOI will be created/registered after the submitter publishes the dataset.' | https://zenodo.org/ | As a part of OpenAIRE (Open Access Infrastructure for Research in Europe), Zenodo is hosted at the Data Center at the European Organization for Nuclear Research (CERN), an Intergovernmental Organization (IGO). Data protection complies with CERN’s Operational Circular 11 (OC11). |
| **Figshare** | Metadata is recommended for the study (title, research category, item type, description, keywords, funding, resource title/DOI, references, research institutions, contact email), in particular a license type for data reuse. | <https://help.figshare.com/article/guide-to-sharing-data-on-figshare-plus> | Data quota for free usage of figshare is 20GB, with 20GB file size restriction and a 100 project cap. With the commercial version of Figshare+, users can upload files greater than 20GB and the storage allowance is up to TB. |
