# Supplementary material for: Genotype and phenotype data standardization, utilization and integration in the big data era for agricultural sciences
Source: Database (Oxford). 2023 Dec 11;2023:baad088. doi: 10.1093/database/baad088 (PMC10712715; doi:10.1093/database/baad088)
Supplement: baad088_Supp [file baad088_supp.zip › suppl_data/SuppTable2.docx]

| **Supplementary Table 2. A list of sequence specific data resources.** | | |  |  |  |
| --- | --- | --- | --- | --- | --- |
| **Database** | **Description** | **Note** | **Current release** | **Link** | **Reference** |
| **PlantProm** | Database of plant promoters. | Current release (2016.03) contains verified transcription start sites (TSS) from 576 plant promoter regions. | 2016 | http://www.softberry.com/berry.phtml?topic=plantp_2016.03&subgroup=plantprom&group=data&no_menu=on | Shahmuradov IA, Gammerman AJ, Hancock JM, Bramley PM, Solovyev VV (2003) PlantProm: a database of plant promoter sequences. Nucleic Acids Res., 31: 114-117 (PMID: 12519961). |
| **Ppdb** | Plant proteome database (Ppdb) | Arabidopsis and maize | 2004 | http://ppdb.tc.cornell.edu/ | Joint project between Klaas J. van Wijk Lab of Cornell University and the Computational Biology Service Unit of Cornell Life Sciences Core Laboratories Center. |
| **PlantPan** | Plant transcription factors (TF) | PlantPAN 3.0 contains 17,230 TF and 4,703 matrices of TF binding sites among 78 plant species. | 2019 | http://plantpan.itps.ncku.edu.tw/ | Chow, C. N., Lee, T. Y., Hung, Y. C., Li, G. Z., Tseng, K. C., Liu, Y. H., ... & Chang, W. C. (2019). PlantPAN3. 0: a new and updated resource for reconstructing transcriptional regulatory networks from ChIP-seq experiments in plants. Nucleic acids research, 47(D1), D1155-D1163. |
| **PlantCare** | Plant TF database. | PlantCARE contains 435 plant transcription sites from 149 monocots, 281 dicots and 5 other plants. It is free to use for academic users. |  | http://bioinformatics.psb.ugent.be/webtools/plantcare/html/ | Lescot, M., Déhais, P., Thijs, G., Marchal, K., Moreau, Y., Van de Peer, Y., ... & Rombauts, S. (2002). PlantCARE, a database of plant cis-acting regulatory elements and a portal to tools for in silico analysis of promoter sequences. Nucleic acids research, 30(1), 325-327. |
| **Rfam** | A database of RNA sequence families of structural RNAs. | New families with functional classification and supporting evidence for a secondary structure can be submitted by email. | 2022 | https://rfam.org/submit_alignment | S. Griffiths-Jones, A. Bateman, M. Marshall, A. Khanna and S.R. Eddy Nucleic Acids Research (2003) 31(1):p439-441 S. Griffiths-Jones, S. Moxon, M. Marshall, A. Khanna, S.R. Eddy, A. Bateman Nucleic Acids Research (2005) Database Issue 33:D121-D124 |
| **miRBase** | A database of miRNA. | Recommended submitting new hairpin and novel miRNAs through mirbase@manchester.ac.uk | 2019 | https://www.mirbase.org/ | miRBase: from microRNA sequences to function. Kozomara A, Birgaoanu M, Griffiths-Jones S. Nucleic Acids Res 2019 47:D155-D162 miRBase: integrating microRNA annotation and deep-sequencing data. Kozomara A, Griffiths-Jones S. Nucleic Acids Res 2011 39:D152-D157 miRBase: tools for microRNA genomics. Griffiths-Jones S, Saini HK, van Dongen S, Enright AJ. Nucleic Acids Res 2008 36:D154-D158 |
| **RNAcentral** | The non-coding RNA (ncRNA) sequence collection | Formed from 57 Expert Databases, eveloped by EBI and supported by Wellcome Charity fund and BBSRC (Biotechnology and Biological Sciences Research Council in UK). | 2023 | https://rnacentral.org/ | "RNAcentral: a comprehensive database of non-coding RNA sequences." Nucleic acids research 45, no. D1 (2017): D128-D134. "RNAcentral: a hub of information for non-coding RNA sequences." Nucleic Acids Research 47, no. D1 (2019): D221-D229. "RNAcentral 2021: secondary structure integration, improved sequence search and new member databases." Nucleic acids research 49, no. D1 (2021): D212-D220. |
| **PlantGDB** | Plant Genomics Database. | Originally NSF-funded (IOS-1221984). Grant ended in 2015. With protein, expressed sequence tag (EST), genomics, cDNA, mRNA and other sequences collected from multiple plant genus. | 2015 | https://www.plantgdb.org/ | [Dong, Q., Schlueter, S.D. & Brendel, V. (2004) PlantGDB, plant genome database and analysis tools. Nucl. Acids Res. 32, D354-D359. [PubMed ID: 14681433 A comprehensive publications are listed at https://www.plantgdb.org/site/publications.php](https://www.plantgdb.org/site/publications.php) |
| **DeepBlue** | Epigenetic data | EWAS Data Hubcatalog; EWASdb; EWAS atlas |  | <https://deepblue.mpi-inf.mpg.de/deepblue_overview.php> | Albrecht,F., List,M., Bock,C. and Lengauer,T. (2016) DeepBlue epigenomic data server: programmatic data retrieval and analysis of epigenome region sets. Nucleic Acids Research, doi:10.1093/nar/gkw211 |
| **Plant Regulomics Portal (PRP)** | DNA methylation, non-coding RNA, histone modification data for Arabidopsis thaliana, soyabean, rice and maize | PRP integrates curated published data from various datatypes: sequencing data (RNA, methylome, sRNA, miRNA), histones, repeats, gene expression and pathways, etc. | * | https://scbb.ihbt.res.in/PRP | Panzade, G., Gangwar, I., Awasthi, S., Sharma, N., & Shankar, R. (2019). Plant Regulomics Portal (PRP): A comprehensive integrated regulatory information and analysis portal for plant genomes. Database, 2019. |
| **CoGE** | Comparative Genomics | Online tool for homologous genomics study. Allow adding new genomes for comparative genomics study. | 2023 | <https://genomevolution.org/coge/> | [Lyons, E., & Freeling, M. (2008). How to usefully compare homologous plant genes and chromosomes as DNA sequences. The Plant Journal, 53(4), 661-673. https://genomevolution.org/wiki/index.php/Contact_Page#How_to_cite_CoGe.3F](https://genomevolution.org/wiki/index.php/Contact_Page#How_to_cite_CoGe.3F) |
| **GreenPhylDB v5** | Pangenome | The version 5.1 contains now a catalogue of clusters / gene families combining 19  pangenomes  (e.g. rice, maize, banana, grape, cacao) and 27 reference genomes for a total of 46 species | 2021 | https://www.greenphyl.org/ | Conte, M. G., Gaillard, S., Lanau, N., Rouard, M., & Périn, C. (2007). GreenPhylDB: a database for plant comparative genomics. Nucleic acids research, 36(suppl_1), D991-D998. Conte, M., Laporte, M. A., Périn, C., & Rouard, M. (2009). GreenPhylDB: A gene family database for plant functional genomics. Nature Precedings, 1-1. Guignon, V., Toure, A., Droc, G., Dufayard, J. F., Conte, M., & Rouard, M. (2021). GreenPhylDB v5: a comparative pangenomic database for plant genomes. Nucleic Acids Research, 49(D1), D1464-D1471. |
| * Unknown: No database release or update information is described at the website. |  |  |  |  |  |
