# Supplementary material for: Genotype and phenotype data standardization, utilization and integration in the big data era for agricultural sciences
Source: Database (Oxford). 2023 Dec 11;2023:baad088. doi: 10.1093/database/baad088 (PMC10712715; doi:10.1093/database/baad088)
Supplement: baad088_Supp [file baad088_supp.zip › suppl_data/SuppTable3.docx]

| Supplementary Table 3 . A list of public crop community databases with data types, metadata, submission format, and URL for data submission | | | | |  |  |
| --- | --- | --- | --- | --- | --- | --- |
| **Species/Crop** | **Database** | **Database URL** | **Data types that can be submitted** | **Metadata summary** | **Submission Format** | **URL for list of meta data/data submission files/online form** |
| Arabidopsis | TAIR | <https://www.arabidopsis.org/> | gene function annotation | author (ORCID), GO, evidence code | online submission system (GOAT) | <https://goat.phoenixbioinformatics.org/> |
|  |  |  | gene family |  | XLS | <https://www.arabidopsis.org/submit/genefamily_submission.jsp> |
|  |  |  | pathway |  | XLS | <https://www.arabidopsis.org/submit/pathway_submission.jsp> |
|  |  |  | mutant phenotype |  | XLS, tab delimited text file | <https://www.arabidopsis.org/submit/phenotype_submission.jsp> |
|  |  |  | marker/allele |  | XLS | <https://www.arabidopsis.org/submit/marker_submission.jsp> |
| Cassava | CassavaBase | <https://www.cassavabase.org/> | accession metadata | uniquename, synonyms, species, + user configurable attributes | XLSX | <https://cassavabase.org/breeders/upload> |
|  |  |  | seedlots | uniquename, storage location, assay information | XLSX | <https://cassavabase.org/breeders/upload> |
|  |  |  | pedigree data | female parent, male parent, type | CSV | <https://cassavabase.org/breeders/upload> |
|  |  |  | field layouts | plot ids, accession, field coords, replicate, etc. | XLSX | <https://cassavabase.org/breeders/upload> |
|  |  |  | phenotyping data | observation unit id, trait, trait value, operator, timestamp | XLSX, fieldbook app | <https://cassavabase.org/breeders/upload> |
|  |  |  | genotyping plate data | source observation unit id, plate coords, operator, timestamp | XLSX, coordinate app | <https://cassavabase.org/breeders/upload> |
|  |  |  | genotyping data | VCF file metadata | XLSX (InterTek format), VCF | <https://cassavabase.org/breeders/upload> |
|  |  |  | locations | location name, county, country, geojson, type, etc. | XLSX | <https://cassavabase.org/breeders/upload> |
|  |  |  | crossing data | #pollinations, #seeds, #fruit, etc. location, operator, timestamp | XLSX, Intercross, btract | <https://cassavabase.org/breeders/upload> |
|  |  |  | analysis results | analysis model, algorithm, input data, results | XLSX | <https://cassavabase.org/breeders/upload> |
| Citrus | Citrus Genome Database | <https://www.citrusgenomedb.org/> | whole genome sequence and annotation | species, cultivar/accession, software, materials/methods, publication | GFF, FASTA, TXT | <https://www.citrusgenomedb.org/tgg/data_submission> |
|  |  |  | marker/allele | species/germplasm, polymorphism type, genome/genetic position | XLS | <https://www.citrusgenomedb.org/tgg/data_submission> |
|  |  |  | genetic maps | experiments, germplasm, marker | XLS | <https://www.citrusgenomedb.org/tgg/data_submission> |
|  |  |  | QTL | experiments, markers, germplasm, phenotype ontology terms, statistical values | XLS | <https://www.citrusgenomedb.org/tgg/data_submission> |
| Citrus / Diaphorina citri / Ca. Liberibacter asiaticus | Citrus Greening | <https://www.citrusgreening.org/> | accession metadata | uniquename, synonyms, species, + user configurable attributes | XLSX | <https://citrusgreening.org/breeders/upload> |
|  |  |  | seedlots | uniquename, storage location, assay information | XLSX | <https://citrusgreening.org/breeders/upload> |
|  |  |  | pedigree data | female parent, male parent, type | CSV | <https://citrusgreening.org/breeders/upload> |
|  |  |  | field layouts | plot ids, accession, field coords, replicate, etc. | XLSX | <https://citrusgreening.org/breeders/upload> |
|  |  |  | phenotyping data | observation unit id, trait, trait value, operator, timestamp | XLSX, fieldbook app | <https://citrusgreening.org/breeders/upload> |
|  |  |  | genotyping plate data | source observation unit id, plate coords, operator, timestamp | XLSX, coordinate app | <https://citrusgreening.org/breeders/upload> |
|  |  |  | genotyping data | VCF file metadata | XLSX (InterTek format), VCF | <https://citrusgreening.org/breeders/upload> |
|  |  |  | locations | location name, county, country, geojson, type, etc. | XLSX | <https://citrusgreening.org/breeders/upload> |
|  |  |  | crossing data | #pollinations, #seeds, #fruit, etc. location, operator, timestamp | XLSX, Intercross, btract | <https://citrusgreening.org/breeders/upload> |
|  |  |  | analysis results | analysis model, algorithm, input data, results | XLSX | <https://citrusgreening.org/breeders/upload> |
| Cotton | CottonGen | <https://www.cottongen.org/> | whole genome sequence and annotation | species, cultivar/accession, software, materials/methods, publication | GFF, FASTA, TXT | <https://www.cottongen.org/data/submission> |
|  |  |  | gene function annotation | author (ORCID), GO, evidence code | XLS | <https://www.cottongen.org/data/submission> |
|  |  |  | genotype evaluation data | experiments, markers, germplasm, marker genome position | XLS, VCF | <https://www.cottongen.org/data/submission> |
|  |  |  | mutant phenotype/image | trait locus name, species | XLS, image file | <https://www.cottongen.org/data/submission> |
|  |  |  | marker/allele | species/germplasm, polymorphism type, genome/genetic position | XLS | <https://www.cottongen.org/data/submission> |
|  |  |  | genetic maps | experiments, germplasm, marker | XLS | <https://www.cottongen.org/data/submission> |
|  |  |  | QTL | experiments, markers, germplasm, phenotype ontology terms, statistical values | XLS | <https://www.cottongen.org/data/submission> |
|  |  |  | GWAS | experiments, markers, germplasm, phenotype ontology terms, statistical values | XLS |  |
|  |  |  | phenotype evaluation | experiments, germplasm, phenotype ontology terms, environment | XLS |  |
| Cucurbit | Cucurbit Genomics | <http://cucurbitgenomics.org/> |  |  |  |  |
| Forest trees | TreeGenes | [https://treegenesdb.org](https://treegenesdb.org/) | whole genome sequence and annotation | species, cultivar/accession, software, materials/methods, publication |  |  |
|  |  |  | genotype evaluation data | experiments, markers, marker genome position |  |  |
|  |  |  | marker/allele | species, polymorphism type, genome/genetic position |  |  |
|  |  |  | genetic maps | experiments, marker |  |  |
|  |  |  | phenotype evaluation | experiments, phenotype ontology terms, environment |  |  |
|  | Hardwood Genomics | <http://www.hardwoodgenomics.org/> |  |  |  |  |
| Grains | GrainGenes | [https://wheat.pw.usda.gov](https://wheat.pw.usda.gov/GG3/) | whole genome sequence and annotation |  |  |  |
|  |  |  | marker/allele | species/germplasm, polymorphism type, genome/genetic position | XLS | <https://wheat.pw.usda.gov/GG3/submit_to_graingenes> |
|  |  |  | genetic maps | experiments, germplasm, marker | XLS | <https://wheat.pw.usda.gov/GG3/submit_to_graingenes> |
|  |  |  | QTL | experiments, markers, germplasm, phenotype ontology terms, statistical values | XLS | <https://wheat.pw.usda.gov/GG3/submit_to_graingenes> |
|  | Gramene | <https://www.gramene.org/> | Whole genome sequence and annotation from Ensembl Plants |  | FASTA, GFF, GTF |  |
|  |  |  | Variation from Ensembl Plants |  | VCF |  |
|  |  |  | Whole gene alignments and protein gene tree analysis from Ensembl Compara |  |  |  |
|  |  |  | Comparative genomics - Orthologs, paralogs from Ensembl Compara tree |  |  |  |
|  |  |  | Gene expression from EBI expression atlas |  |  |  |
|  |  |  | Ontology from Planteome |  |  |  |
|  |  |  | Pathways from Gramene's Plant Reactome |  |  |  |
